# Legislation and current developments in adverse drug reaction reporting in Mongolia: how far are we?

**DOI:** 10.1186/s40545-021-00298-8

**Published:** 2021-03-08

**Authors:** Zuzaan Zulzaga, Erdenetuya Myagmarsuren, Herman J. Woerdenbag, Eugene P. van Puijenbroek

**Affiliations:** 1 Association of Mongolian Pharmacy Professionals, Apart # 88-33, 25th khoroo, Bayanzurkh District, Ulaanbaatar, Mongolia; 2 Department of Clinical Pharmacy and Management, School of Pharmacy, Mongolian National University of Medical Sciences, Ard Ayush street, 6th khoroo, Bayangol district, P.O.Box-188, Ulaanbaatar, Mongolia; 3 Department of Pharmacy, Mongolian Japan Hospital of Mongolian National University of Medical Sciences, Botanik street, 12nd khoroo, Bayanzurkh district, Ulaanbaatar, Mongolia; 4 Department of Pharmaceutical Technology and Biopharmacy, University of Groningen, Antonius Deusinglaan 1, 9713 AV Groningen, The Netherlands; 5 Pharmacovigilance Centre Lareb, Goudsbloemvallei 7, 5237 MH ′s-Hertogenbosch, The Netherlands; 6 Department of PharmacoTherapy, - Epidemiology & -Economics, University of Groningen, Antonius Deusinglaan 1, 9713 AV Groningen, The Netherlands

**Keywords:** Adverse drug reaction, Mongolia, Medicines safety

## Abstract

Monitoring adverse drug reactions is a vital issue to ensure drug safety and to protect the general public from medication-related harmful effects. In order to properly monitor drug safety, a regulatory system needs to be in place as well as an infrastructure that allows for analyzing national and international safety data. In Mongolia, adverse drug reaction (ADR) reporting activities have been implemented in the past decade. During this period, the basic structure and legal basis of an adverse drug reaction monitoring system was established. Because of the fragmented but growing healthcare system and the complexity of pharmaceutical issues in Mongolia, a sustainable process for the development of the adverse drug reaction reporting system is a key issue. The aim of this article is to disclose the Mongolian situation for the rest of the world and to share experiences on how an ADR reporting system can be developed towards a higher and more advanced level to contribute to both national and international drug safety issues. In this article, we review the features of the Mongolian health care and pharmaceutical systems, as well as the current development of the adverse drug reaction reporting system.

## Background

Modern medicines have significantly changed the way in which diseases can be managed and controlled. However, adverse drug reactions (ADR) may occur. They form a cause of illness, disability and even death that should be prevented as much as possible. In North America and Europe, adverse drug reactions have been reported to be a significant cause of morbidity and mortality [1, 2]. In addition, some research showed that among patients who experienced an ADR, approximately half of them are preventable [3].

In order to prevent or reduce unnecessary harm to patients and thus to improve public health, procedures for evaluating and monitoring the safety of medicines in clinical use are vital [4]. Improving drug safety and preventing the population from drug related problems is a global concern. Health requirements and the use of medicines vary considerably between countries for many reasons, including different burdens of disease, economic, ethnic, cultural and dietary factors, and in the level of development of a regulatory system for medicines. Decisions concerning the effectiveness and safety of a medicinal product need to be considered in each country’s specific context [5].

Asia’s pharmaceutical market is generally dominated by generic drugs. Asia is the fastest growing pharmaceutical market in the world, offering important opportunities for drug development and marketing. Consequently, pharmaceutical regulations in this region are rapidly gaining attention among pharmaceutical companies worldwide [6]. Pharmacovigilance in Asia has become an important public health issue as regulators, drug manufacturers, consumers, and health care professionals are faced with a number of challenges. Pharmacovigilance goes beyond the submission of case reports of suspected adverse effects of medicines. It involves complex processes including the need to monitor the safety of medicines throughout their lifecycle and to manage identified real and potential risks. This will ultimately improve both the knowledge and quality of health care [7, 8].

The legal basis of adverse drug reaction reporting activities in Mongolia is considered rather well and has been enforced since 2006. There are a number of achievements and challenges on regulating and ensuring the safety of medicines that are comparable to other Asian countries. However, little is known about this outside Mongolia, on an international level. Only a few studies with limited scope on this topic have been published in national professional journals and in the form of conference abstracts [8–12].

This article further aims to share current developments and challenges regarding the characteristics of the adverse drug reaction reporting system in Mongolia. Sharing experiences can be helpful for the development of countries in a similar situation. After briefly introducing the Mongolian demographic situation, we give an overview of the Mongolian health care system. And, we discuss the organizational structure of the pharmaceutical sector including adverse drug reaction reporting system and marketing authorization of medicinal products according to the Mongolian situation. Finally, we discuss the challenges and give our opinion towards the future direction.

## Demographic background

Mongolia is located in East-Asia and with estimated area of 1.6 million km^2^ and around 3.2 million inhabitants (in 2020). Life expectancy at birth was estimated to be 70.2 years in 2018, indicating that longevity has increased by approximately 6 years over the last 20 years. Gender difference in life expectancy at birth has also expanded reaching 9.7 years in 2018. It is obvious that there has been a shift from rural to urban population over the years (Table 1) [13].

**Table 1:** Demographic and health indicators, selected years ( adopted from Health Indicators of Mongolia, 2018)

Indicator	1990	2010	2015	2018
Total population (thousands)	2149.2	2780.7	3057.7	3238.4
Urban population (%)	54.6	63.3	68.0	67.8
Rural population (%)	45.4	36.7	32.0	32.2
Age groups (%)				
0–15	41.5	27.3	29.6	31.2
15–64	54.4	68.8	66.6	64.7
Above 65	4.1	3.9	3.8	4.1
Life expectancy at birth (years)				
Total	63.7	68.1	69.9	70.2
Male	60.3	64.9	66.0	66.1
Female	67.6	72.3	75.8	75.8

## The Mongolian health care system

The health care system of Mongolia consists of state-owned, private and mixed-owned health facilities that are in charge of public health, medical care service, pharmaceuticals supply, health education, research and training. The delivery of health services is challenged by the country’s extremely low population density over a large territory[14].

Family health centers (FHCs) are groups of primary care physicians that provide primary healthcare services in the capital Ulaanbaatar, in province centers, and in other cities. For the last few years, the workload of family doctors has increased substantially in Ulaanbaatar due to the city’s increased population through internal migration. In rural areas primary health care is provided through the soum health centers (a soum is an administrative unit in a Mongolian province). A soum health center also operates 10–15 beds for inpatient care, basic surgery, and delivery services, because of the geographical distances and a large coverage area [14, 15].

At the secondary level, specialized ambulatory care and inpatient care are provided by the province and by the district general hospitals of Ulaanbaatar city as well as by district ambulatory and private hospitals.

Tertiary level care is provided through the specialized state hospitals and specialty centers which are located in Ulaanbaatar and by the Regional diagnostic and treatment centers provide specialized tertiary level referral, diagnostic and treatment services to the population outside the capital. Some ministries have their own hospitals which provide secondary level services to their workers [15].

Since the governmental approval of health sector privatization in 2001, the private health sector has become a strong competitor to government hospitals in terms of human resource capacity, user-friendly care, and equipment. The private sector provides mainly secondary level specialty services. At present, the private sector dominates in the areas of dentistry, internal medicine, obstetrics and gynecological care, traditional medicine, and high-tech laboratory services [15].

## Overview of the pharmaceutical sector

### Organizational structure of the pharmaceutical sector

Mongolia is one of the very few countries in the world without a nationwide regulatory agency for the control of medicinal products yet. Therefore, drug regulatory functions are undertaken by different organizations. This makes it difficult to coordinate them and leads to hampered efficiency [15].

**Fig. 1:**
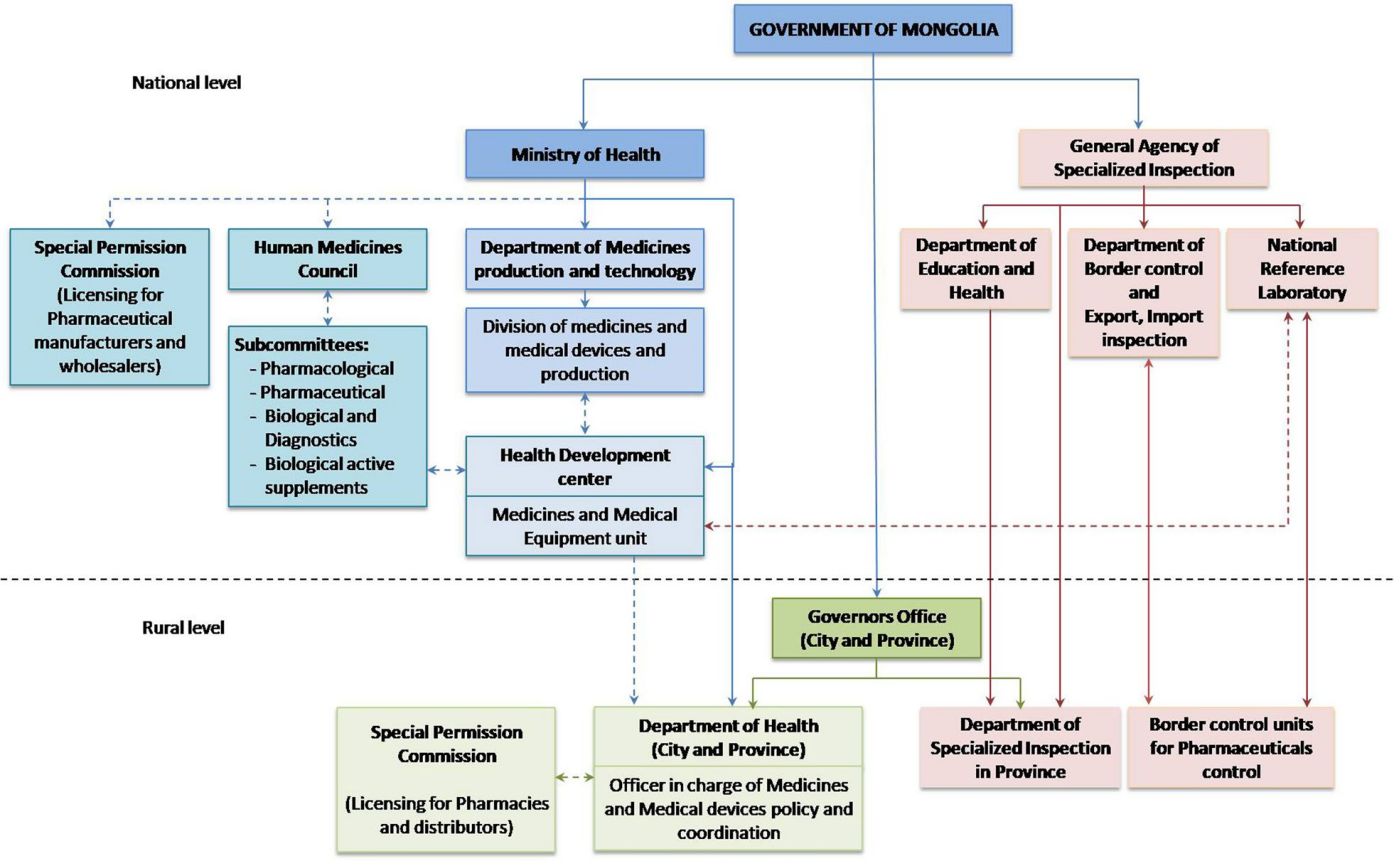
Governance of pharmaceutical sector of Mongolia. The Ministry of Health (MOH) is responsible for oversight of the main functions of the pharmaceutical sector policy and regulation at the national level. The Health Development Center (HDC) and its Medicines and Medical Equipment unit is a professional organization affiliated with the MOH, which provides support for policy formulation and technical capacity strengthening in the areas of pharmaceutical system. Both the Human Medicines Council and Special Permission Commission of the MOH are operational without permanent staff (dashed arrows). General Agency for Specialized Inspection (GASI) is the government regulating agency in charge of monitoring the implementation of state regulations and standards, including those related to the health and pharmaceutical system. The National quality control laboratory is affiliated with the GASI and aims to offer a full service for testing all pharmaceuticals. At the regional level, MOH and GASI make control and collaboration with the Governors’ office and its departments of the city and province

The Ministry of Health (MOH), the Division of Medicines and Medical Devices and Production are responsible for oversight of the main functions of the pharmaceutical sector policy, for regulation and supervision and to ensure the implementation of regulatory functions (Fig. 1). The MOH has Human Medicines Council and Special Permission Commission, consisting of experts in the pharmaceutical and medical field and representatives of the professions. The Human Medicines Council makes professional decisions, leads the pharmaceutical sector, particularly regarding the development of standards, guidelines and procedures, including medicines registration. The Special Permission Committee of the MOH grants approval for manufacturing, importing and selling medicines in Mongolia. The Health Development Center and its Medicines and Medical Equipment Unit is responsible as secretariat for the Human Medicines Council including the registration of medicines and diagnostics, issuing import and export licenses, monitoring and reporting adverse drug reactions, monitoring medicines marketing and advertisement, promoting rational use of medicines and developing a national pharmacopeia and various standards. The General Agency for Specialized Inspection (GASI) is the governmental regulating agency which is in charge of monitoring the implementation of state regulations and standards, including those related to the health and pharmaceutical systems. Through the Department of Education and Health, the Department of Border Control and Export, the Import inspection and the National Reference Laboratory compliance with major laws and legislation is ensured, related to quality assurance and inspection of drug distribution at the national level [15, 16].

### Marketing authorization of medicines

The Mongolian pharmaceutical sector is completely dependent on private manufacturing, on pharmaceutical wholesalers and on pharmacies. With accelerating growth of the private sector, the drug supply business has proven to be very profitable. Before a drug becomes available in the country it should first be registered upon agreement and authorization by the Human Medicines Council [15, 16].

**Fig. 2:**
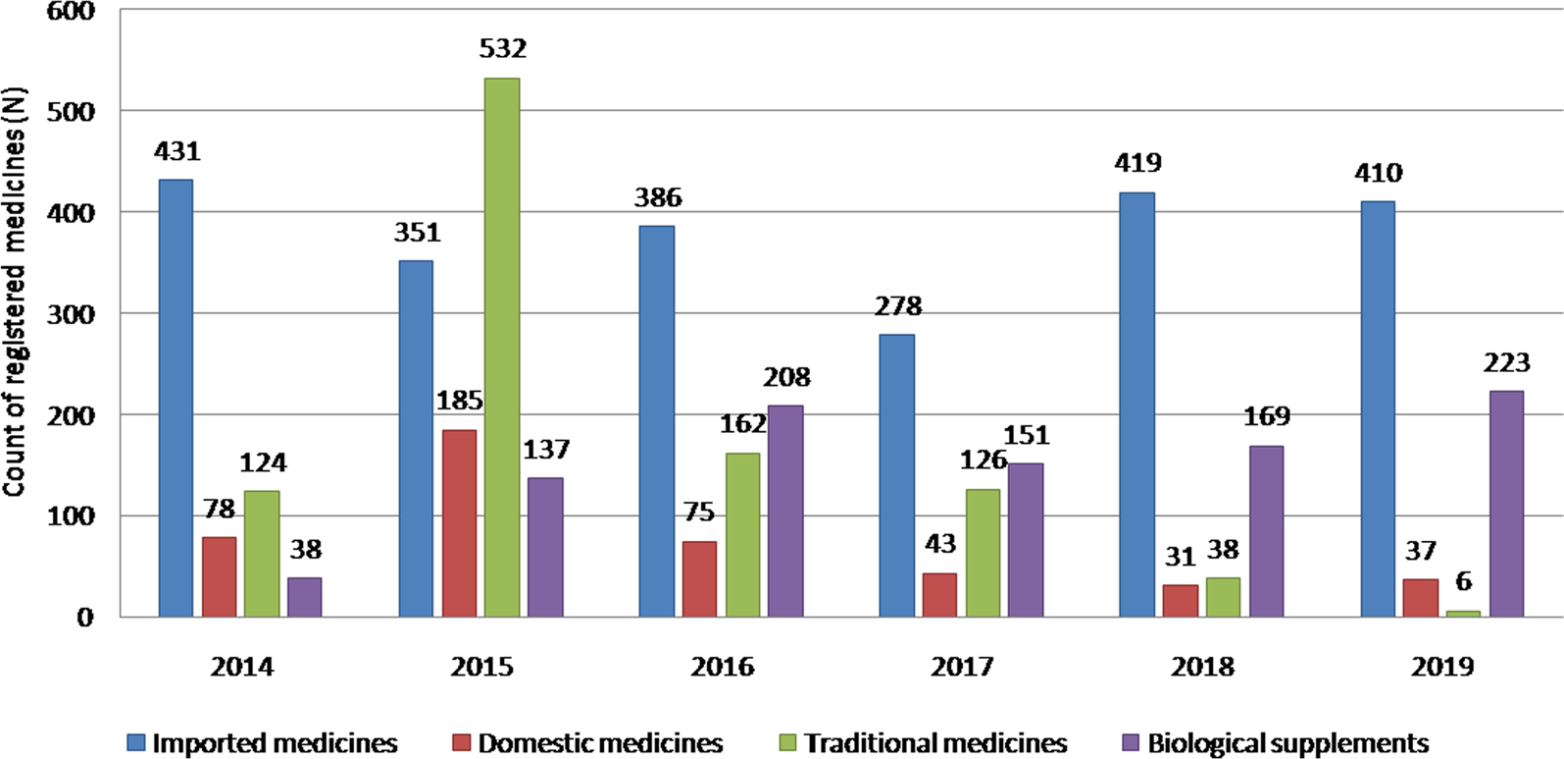
Newly registered medicines, traditional medicines and biological supplements per year during 2014–2019 ( adopted from Health indicators of Mongolia, 2019)

Currently, there are 41 pharmaceutical manufactures, 360 wholesalers and 1729 pharmacies of which 894 are located in the capital city of Ulaanbaatar and 835 in the provinces [17]. By the end of 2019, there were 4558 registered medicines marketed in the Mongolian health care system, of which 25.8% were domestic and 74.2% were imported products from a total of 58 countries. Most imported medicines sold in Mongolia come from the India (12.4%), Korea (9%), Russia (6.4%), Germany (5.8%), Indonesia (4.5%), Slovenia (4.45%), China (3.5%), Ukraine (2.04%) and Belarus (1.7%). The rest is from other countries. Biologically active products and/or locally produced traditional medicines have to go through the same procedures as other pharmaceuticals for registration, licensing and quality assurance (Fig. 2) [18].

## Regulation of the adverse drug reaction reporting system and current developments

Drug safety and adverse drug reporting activities in Mongolia are governed by the Law on Medicines and Medical Devices. The main concept of this legislation is to ensure that good quality, effective and safe medicines are available to the entire Mongolian population. According to the Law on Medicines and Medical Devices, the ensurance of safety of medicines and maintaining a procedure for reporting adverse drug reactions are legislated by the Ministry of Health (MOH). The first Ministerial order for “Adverse drug reaction reporting” was approved in 2006 (No. 183) and was revised twice so far, in 2010 (No. 378) and in 2013 (No. 415) [19–21].

**Fig. 3:**
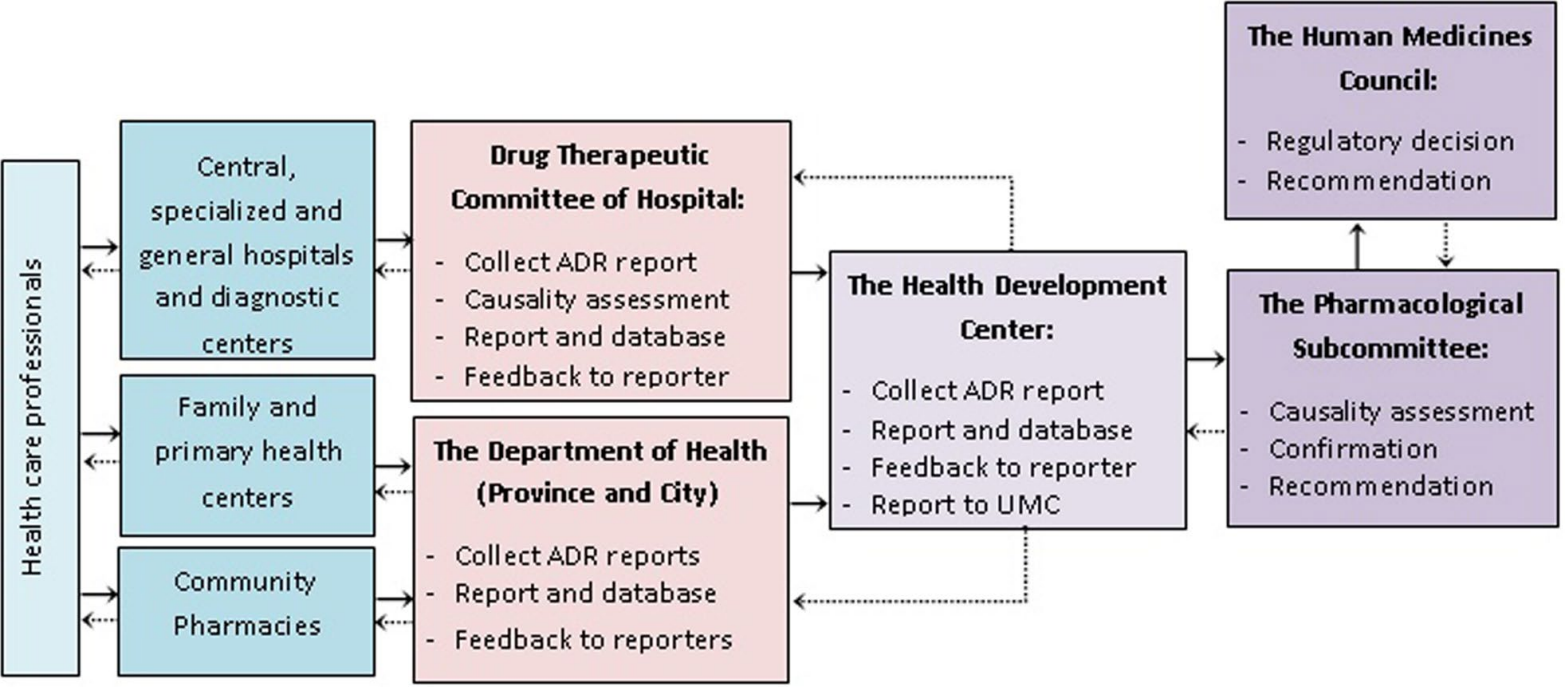
Flow of ADR reporting system in Mongolia and stakeholders involved. A medical professional who first detects an ADR should fill out an ADR reporting form and send it to the own health organization. The central, specialized and general hospitals and diagnostic centers have a Drug Therapeutic Committee that discusses and forwards the information to The Health Development Center (HDC). The Medicines and Medical Equipment unit of the HDC is responsible for drug safety and The Human Medicines Council of the MOH makes decisions. (The straight arrows represent the flow of ADR reporting and the dashed arrows represent the feedback flow.)

Currently the effective ministerial order named “Policy of reporting of Adverse drug reaction and Periodic safety update report” (415/2013) aims to improve drug safety in Mongolia. The main part of this order regulates the managing and monitoring of reporting, collecting and analyzing of suspected adverse drug reactions and the subsequent actions to be taken. The structure of the adverse drug reaction reporting system in Mongolia is shown in Fig. 3.

A pharmacist employed at the Medicine and Medical Equipment unit is responsible for drug safety and collects the submitted adverse drug reaction reports from the health centers nationwide. This pharmacist organizes meetings with the Pharmacological Subcommittee. This Subcommittee discusses reported adverse drug reaction cases and evaluates relations between the adverse drug reaction case and the suspected medicine, the type of adverse drug reaction and signal detection and formulates recommendations to improve drug safety. The Human Medicines Council of MOH receives the recommendation from the Pharmacological Subcommittee and defines actions needed to be taken and makes decisions regarding actions to be taken related to drug safety issues.

According to the national policy, all health care professionals such as medical doctors, pharmacists and nurses are mandatory to report adverse drug reactions experienced in their work in health organizations and pharmacies. There is no direct patient adverse drug reaction reporting form and method yet. However, the patients who experience adverse drug reaction should address their medical professionals.

Currently, the “Adverse drug reaction reporting form” that is used to report suspected adverse drug reactions is based on the “CIOMS-I form” (The Council for International Organizations of Medical Sciences) [22]. It is designed to cover all required information about the patient and the suspected medicine, start and stop time of the medicine, onset time and symptoms of adverse drug reaction, outcome and actions taken to counteract the adverse drug reaction. Besides, it is required to fill in information about other medicines used concomitantly with the suspected medicine as well as contact details of reported person.

The reporting timeline of adverse drug reaction cases is within 7 days for serious adverse drug reactions, according to the definition of CIOMS working group [22]. Any other less life-threatening adverse drug reaction cases should be reported within 14 days to the Health Development Center.

In the period 2012–2015, “The 4th Health Sector Development Project” was implemented in Mongolia with financial and technical support from the Asian Development Bank and from the World Health Organization in order to strengthen the drug safety in Mongolia. The international experts of the project analyzed the drug regulatory and drug safety system of Mongolia and recommended to establish and to improve pharmacovigilance and an adverse drug reaction reporting system [23]. Training for health professionals about the understanding and importance of adverse drug reaction reporting was developed and a handbook for health professionals and marketing authorization holders was provided [24, 25].

Since 2015, the Health Development Center publishes official data from pharmaceutical sectors as well as information on drug safety to make it available for health professionals. Table 2 shows published data related to the adverse drug reaction reporting activities in Mongolia over the period 2013–2019. However, it only gives information as to the number of submitted adverse drug reaction case reports and the number of reporting health organizations but no details about a detailed analysis of adverse drug reaction cases, detection of new adverse drug reaction signals and actions taken [18].

**Table 2:** Number of reported adverse drug reaction reports from various health organizations in Mongolia ( adopted from Indicators of the Pharmaceutical sector of Mongolia, 2019) [18]

Indicator		2013	2014	2015	2016	2017	2018	2019
Hospitals and pharmacies of Ulaanbaatar	Number of health organizations reporting	14	11	5	16	9	15	21
	Number of ADR reports	111	98	44	117	59	95	74
Hospitals and pharmacies of province	Number of health organizations reporting	7	7	8	6	7	2	6
	Number of ADR reports	66	60	24	106	27	7	16
Total	Number of health organizations, reporting	21	18	13	22	16	17	27
	Number of ADR reports	177	158	68	223	86	102	90

In 2012, Mongolia became an associate member of the UMC/WHO collaborate center for International Drug Monitoring. However, Mongolia is not a full member and does not forward adverse drug reaction reports to international adverse drug reaction databases yet [26].

## Challenges for the future

For the effective and proactive development of an adverse drug reaction reporting system in a country, governmental commitment and financial support, as well as a responsible infrastructure are essential [27]. Also, in Mongolia, sustainable process development of the adverse drug reaction reporting system is a key issue. In fact, there is only one position (a pharmacist) in charge of adverse drug reaction reporting and drug safety. Due to the high workload and lack of other experienced professionals, there are continuous changes in staffing among the responsible persons. This is a major problem for developing a stable system.

In Mongolia, adverse drug reaction reporting activities have been implemented in more than 10 years. During this period, the basic structure and the legal basis of the adverse drug reaction monitoring system has been established from the minimum requirements for a functional Pharmacovigilance system as described by the WHO [28]. Awareness and understanding of the importance of adverse drug reaction reporting has been developed among health professionals. The number of adverse drug reaction reports from health care organizations and medical professionals has become sustainable in the recent years, although it varies among the different health care organizations. In order to encourage the healthcare professionals to report adverse drug reactions, it is needed to improve a proactive feedback system and to organize continuous training to improve awareness and knowledge about adverse drug reactions. Also, in academic curricula (medicine, pharmacy) more attention is given to the relevance of adverse drug reaction reporting. In the Mongolian situation, the introduction of an electronic reporting system might facilitate adverse drug reaction reporting activities and the implementation of an appropriate feedback system.

Currently, similar to other many low and middle-income countries, there is no scientific evidence on the local burden of medicine-related harm and information on their preventability is missing [29].

Because of the fragmented healthcare system and the weak regulatory oversight, the pharmaceutical supply chain in resource-limited countries is characterized by poor enforcement of legislation. Under these conditions, substandard, falsified medicines and vaccines can easily penetrate the supply chain [29, 30]. This also holds for the Mongolian health care system and for the pharmaceutical sector. As the pharmaceutical industry, wholesalers and pharmacies expand in the Mongolian benchmark, selling unregistered, substandard and falsified medicines for public use [31, 32] is still a problematic and serious issue.

In addition, there are issues with higher rates and high levels of inappropriate use of antibiotic use and the high level of medical prescribing and supply of injections is a significant potential public health hazard in Mongolia [33–36]. However, the number of clinical trials and public health programs in Mongolia has increased [37–39], but is still insufficiently coordinated in terms of drug safety. This implies that there is a need for the evaluation of the baseline situation and that progress should be made regarding systems and assessment of inputs, processes, outputs, outcomes, and impacts of development policies to adverse drug reaction reporting and ensuring drug safety [40]. Subsequently there is a need to establish and expand the pharmacovigilance system in Mongolia beyond the current adverse drug reaction reporting system and develop additional activities focussed at signal detection and confirmation, risk management and communication. Moreover, Mongolia needs to expand its cooperation with the UMC/WHO, train professionals in the field of drug safety and submit the adverse drug reactions in the international databases and to contribute for the ensuring safety of medicine in the globally.

## Conclusion

In Mongolia, the basic structure and the legal basis of the adverse drug reaction reporting system has been established and developed over the past decade to a certain extent, especially in the form of a voluntary reporting. However, to improve effectiveness of the existing system and to improve drug safety in Mongolia it is urgently needed to address the governmental commitment and the financial support in order to build up a professional organization with experienced human resources and leadership. Finally, an increased involvement of healthcare professionals from public and private sectors, pharmaceutical companies, academic institutions and the public at large is necessary to assure the safe use of drugs.

## Abbreviations

ADR: Adverse drug reaction
CIOMS: The Council for International Organizations of Medical Sciences
DTC: Drug and Therapeutic Committee
FHCs: Family Health Centers
GASI: General Agency for Specialized Inspection
HDC: Health Development Center
MOH: Ministry of Health
UMC: Uppsala Monitoring Center
WHO: World Health Organization
